# Short-Term Psychological and Physiological Changes Following Coffee Aroma Exposure: A Multimodal Assessment

**DOI:** 10.3390/nu18182954

**Published:** 2026-09-09

**Authors:** Moeka Fukatsu, Yukihiro Tsuchiya, Yoshihiro Inoue

**Affiliations:** Faculty of Pharmacy, Showa Pharmaceutical University, Tokyo 194-8543, Japanyatsuchi@ac.shoyaku.ac.jp (Y.T.)

**Keywords:** coffee aroma, olfaction, psychophysiological response, electroencephalography (EEG), mood, heart rate variability (HRV)

## Abstract

**Background/Objectives**: Coffee aroma has been associated with psychological and physiological responses, although these responses have often been examined separately. This exploratory study used a multimodal approach to assess subjective mood, electroencephalographic (EEG) activity, heart rate (HR), and heart rate variability (HRV) following short-term coffee aroma exposure. **Methods**: Twenty healthy adults aged 20–29 years participated in a single-group, within-subject pre–post study and were exposed to coffee aroma for 5 min. Mood was assessed using the Profile of Mood States (POMS). Pre- and post-exposure measurements were compared using paired *t*-tests, with Holm adjustment for the six POMS subscales. Effect sizes, 95% confidence intervals (CIs), and exploratory cross-domain correlations were also examined. **Results**: Total Mood Disturbance decreased following exposure (mean change = −20.54, 95% CI: −30.65 to −10.43; Cohen’s dz = −0.95). After Holm adjustment, Fatigue, Tension–Anxiety, Confusion, and Depression–Dejection remained significantly reduced. The EEG-derived relaxation index increased (mean change = 0.0378, 95% CI: 0.0248–0.0508; dz = 1.36). No statistically significant changes were detected in HR (dz = 0.19) or HRV (dz = 0.13). Exploratory cross-domain analyses identified no significant associations among individual changes in subjective, cortical, and cardiovascular measures. **Conclusions**: Short-term coffee aroma exposure was followed by changes in subjective mood and an EEG-derived index, whereas no significant cardiovascular changes were detected. Multimodal assessment may provide complementary information regarding psychological and physiological changes following coffee aroma exposure, although controlled studies with larger samples are required.

## 1. Introduction

Coffee is one of the most widely consumed beverages worldwide and is valued not only for its taste but also for its characteristic aroma [1,2]. The aroma of coffee is composed of numerous volatile compounds generated mainly during roasting and brewing, and olfactory exposure to coffee has been reported to influence psychological states and physiological activity [3,4]. Previous studies have suggested that coffee aroma may affect mood, cognitive performance, stress-related responses, and brain activity. These findings indicate that the experience of coffee aroma may involve multiple aspects of human psychophysiological function.

Subjective psychological responses to odors can be assessed using standardized mood questionnaires, whereas physiological responses can be examined using measures reflecting different functional domains. Many studies have used self-report questionnaires to evaluate psychological responses to coffee aroma, including the Profile of Mood States (POMS), and some studies reported improvements in mood following exposure to coffee aroma [5]. Electroencephalography (EEG), for example, provides information on cortical electrical activity and has been widely used to investigate psychophysiological responses to fragrances [6]. Changes in EEG frequency components, particularly alpha (α), beta (β), and theta (θ) activity, have been associated with alterations in arousal and relaxation-related states. In contrast, heart rate (HR) and heart rate variability (HRV) provide information regarding cardiovascular and autonomic responses [7,8]. Thus, subjective mood, cortical activity, and cardiovascular measures reflect different aspects of the response to olfactory stimulation.

Previous studies have investigated some of these responses to coffee aroma. Coffee fragrance inhalation has been reported to influence mood, working memory, and salivary cortisol [4], while other studies have examined EEG responses associated with coffee aroma have also been reported [9]. However, previous studies have typically focused on selected response domains or have used different experimental settings. Consequently, it remains difficult to determine how changes observed across these domains appear when assessed concurrently in the same individuals under the same exposure conditions.

Importantly, responses across psychological and physiological domains need not necessarily occur in parallel. Subjective experience, cortical electrical activity, and cardiovascular autonomic measures represent distinct levels of psychophysiological function, and concurrent changes at the group level do not necessarily imply direct correspondence at the individual level or a common underlying mechanism. Assessing these domains within the same participants may therefore provide complementary information and allow a more comprehensive characterization of the changes observed following olfactory exposure without assuming that the different measures represent a single integrated response.

**Figure 1:**
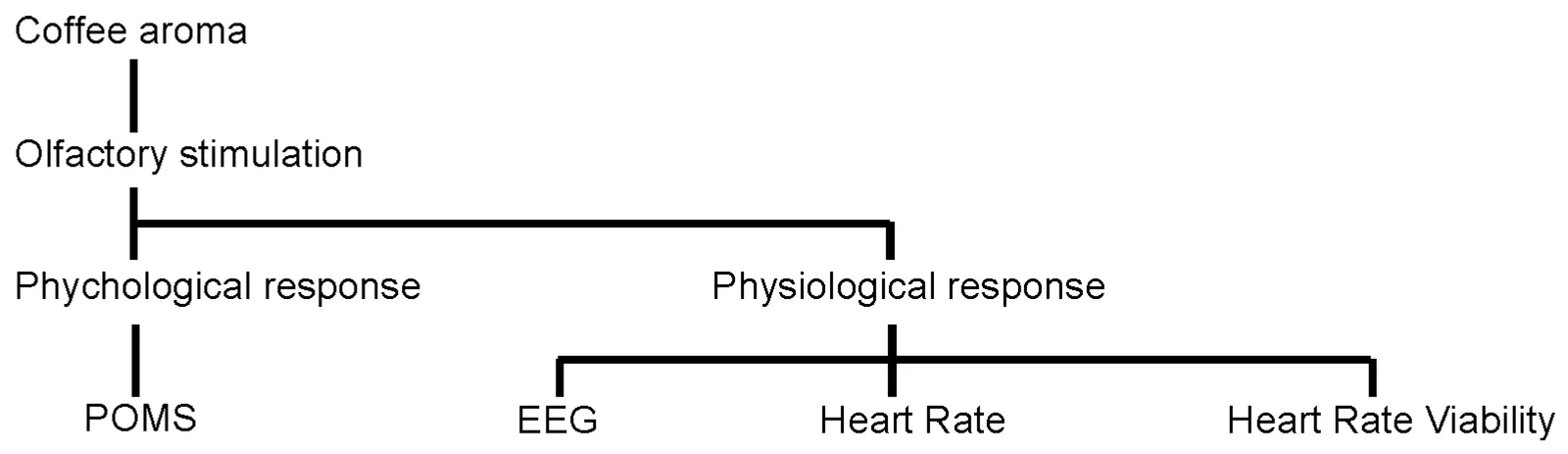
Experimental design. EEG, electroencephalography; POMS, Profile of Mood States.

Therefore, the present exploratory study used a multimodal approach to concurrently assess subjective mood, EEG activity, HR, and HRV before and after short-term coffee aroma exposure in healthy young adults (Figure 1). By examining these response domains within the same participants and experimental setting, we aimed to characterize the psychological and physiological changes observed following coffee aroma exposure and to explore whether individual changes across response domains were associated.

## 2. Materials and Methods

### 2.1. Study Design and Setting

This exploratory study employed a single-group, within-subject pre–post design to examine psychological and physiological changes following short-term coffee aroma exposure. The study was conducted in a laboratory at Showa Pharmaceutical University between March 2024 and March 2026. Psychological and physiological measurements were obtained from the same participants before and after coffee aroma exposure.

The study protocol was approved by the Ethics Committee of Showa Pharmaceutical University (approval No. 2023-3) and was conducted in accordance with the Declaration of Helsinki. Before enrollment, all potential participants received written information explaining the purpose and procedures of the study. They were informed that participation was entirely voluntary, that they could withdraw at any time, and that withdrawal would result in no disadvantage. Written informed consent was obtained from all participants before participation.

### 2.2. Participants

Twenty healthy adults aged 20–29 years were recruited from university students using convenience sampling. The study population consisted of 8 men and 12 women. Participants were eligible if they were healthy adults in the predefined age range. Olfactory function, habitual coffee consumption, coffee preference, and familiarity with coffee aroma were not used as eligibility criteria and were not systematically assessed.

No formal a priori sample-size calculation was performed. The sample size was determined by participant availability within the exploratory nature of the study.

### 2.3. Coffee Preparation and Aroma Exposure

Tsukiyono Monogatari blend F, a Mandheling-based blended coffee developed and marketed by Tatsumi Ryokan (Gunma, Japan), was used in this study. This blend was selected because the production process was fully traceable, which allowed for precise control of the post-roasting period. In addition, the production volume was sufficient to ensure a stable supply of coffee beans of consistent quality throughout the study.

For each experiment, 20 g of ground coffee was prepared by drip extraction using 400 mL of water at 85 °C with a Kalita dripper and paper filter. Extraction was completed in approximately 3 min. These preparation conditions were predefined and standardized specifically for the present experiment rather than adopted from a previously published protocol.

The freshly brewed coffee was placed in a glass coffee server approximately 50 cm from the participant. Participants were exposed continuously to the naturally diffusing coffee aroma for 5 min while breathing normally. The coffee was not reheated during the exposure period to avoid introducing additional changes in aroma caused by continued heating.

**Figure 2:**
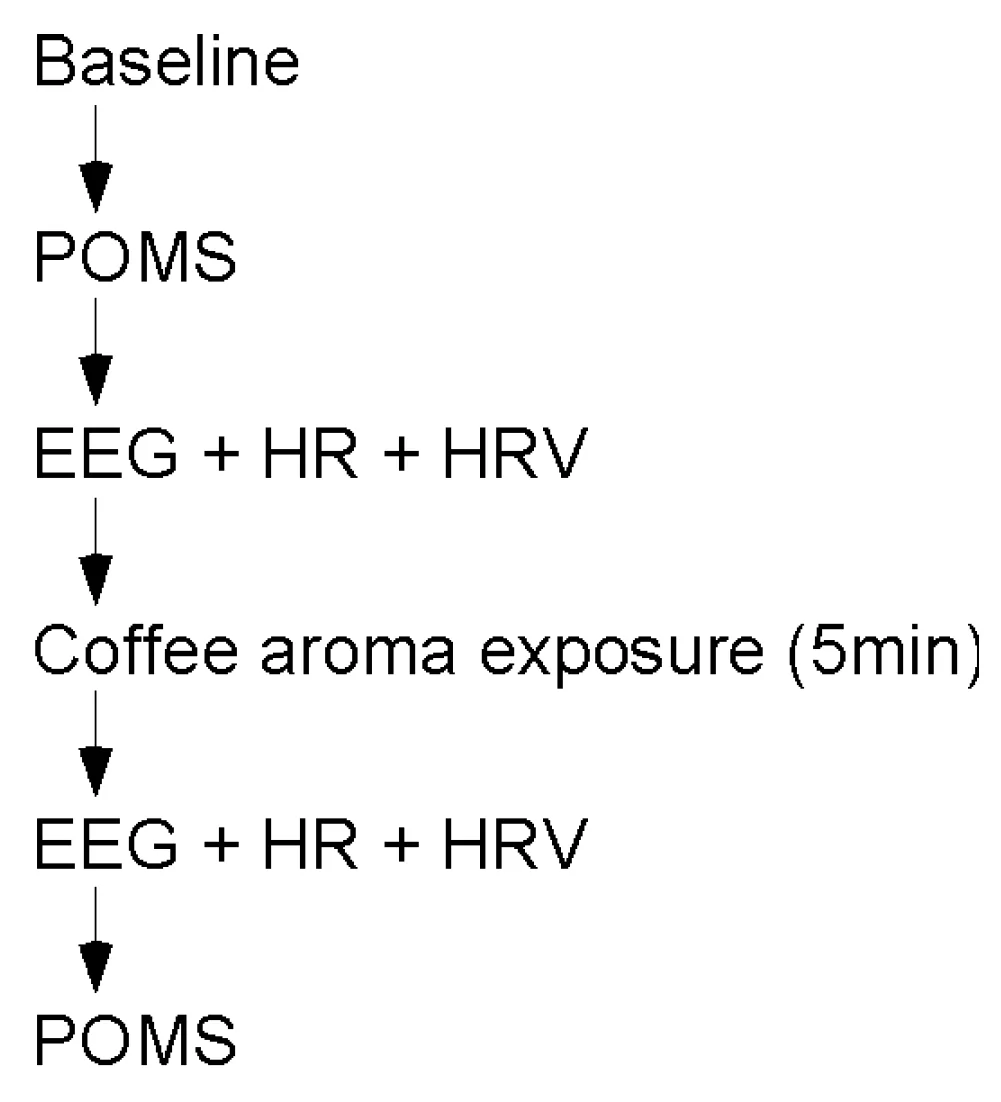
Experimental procedure. POMS and physiological measurements were performed before and after 5 min of coffee aroma exposure. EEG, electroencephalography; HR, heart rate; HRV, heart rate variability.

Experiments were conducted in a 3 × 6 m laboratory room. The room was sufficiently ventilated before each experiment, after which the doors and windows were closed during measurement. No additional odor source was intentionally introduced into the room. Aroma concentration and airflow were not quantitatively monitored, and room temperature and relative humidity were not systematically recorded. The experimental protocol is illustrated in Figure 2.

### 2.4. Psychological Assessment

Mood was assessed immediately before and after coffee aroma exposure using the Japanese adult full-length version of the Profile of Mood States (POMS) [5]. The questionnaire evaluates six mood dimensions: Tension–Anxiety, Depression–Dejection, Anger–Hostility, Vigor, Fatigue, and Confusion. Total Mood Disturbance (TMD) was also calculated according to the standard scoring procedure.

The Japanese adult version of POMS was validated in healthy Japanese adults and has demonstrated acceptable internal consistency across the six mood subscales (Cronbach’s α = 0.779–0.926), together with evidence supporting factorial and criterion-related validity [5].

### 2.5. EEG and Heart Rate Measurements

Electroencephalographic activity was recorded using the Muse 2 headband (InteraXon Inc., Toronto, ON, Canada). Muse 2 is a portable consumer EEG device whose measurement capabilities have been evaluated in previous validation studies [10,11,12].

EEG activity was analyzed using alpha (α), beta (β), and theta (θ) frequency components. An EEG-derived relaxation index was calculated as:

Relaxation index = (α + θ)/(α + β + θ)

based on a previously reported EEG feature-analysis approach [13]. Higher values of this index were interpreted as indicating a greater relative contribution of EEG activity associated with relaxation-related states rather than as a direct measure of relaxation itself.

Heart rate (HR) was also obtained using the Muse 2 device. Measurements used for the pre–post comparison were obtained immediately before and at the end of the 5 min coffee aroma exposure period.

### 2.6. Heart Rate Variability

Heart rate variability (HRV) was measured using a Lanceband wearable device (MedVigilance Inc., Yokohama, Japan). HRV data were analyzed using Crosswell software meijin (Crosswell Co., Ltd., Yokohama, Japan). The low-frequency/high-frequency (LF/HF) ratio was calculated by using the maximum entropy method and used as the HRV index in the present study. LF/HF values obtained before and after coffee aroma exposure were compared.

### 2.7. Statistical Analyses

Statistical analyses were performed using JMP version 13 (SAS Institute Inc., Cary, NC, USA). Descriptive statistics were calculated for all study variables. The normality of paired differences between pre- and post-exposure measurements was assessed using the Shapiro–Wilk test. No statistically significant deviations from normality were detected for the analyzed outcomes (all *p* > 0.05); therefore, pre- and post-exposure measurements were compared using paired *t*-tests.

To address multiple comparisons across the six POMS subscales, the Holm procedure was applied. TMD was treated separately as an overall mood disturbance score rather than as one of the six subscales. Mean differences, 95% confidence intervals (CIs), and Cohen’s dz were calculated to characterize the magnitude and precision of within-subject changes.

Exploratory Pearson correlation analyses were additionally performed using individual pre–post change scores to examine cross-domain associations among TMD, the EEG-derived relaxation index, HR, and HRV. All statistical tests were two-sided, and statistical significance was set at *p* < 0.05.

## 3. Results

### 3.1. Participant Characteristics

A total of 20 healthy adults aged 20–29 years participated in the study, including 8 men and 12 women. All participants completed the pre- and post-exposure assessments and were included in the analysis.

### 3.2. Changes in Mood States

Total Mood Disturbance (TMD) decreased following coffee aroma exposure, with a mean change of −20.54 (95% CI: −30.65 to −10.43; Cohen’s dz = −0.95).

Among the six POMS subscales, reductions were observed in Anger–Hostility, Confusion, Depression–Dejection, Fatigue, and Tension–Anxiety, whereas Vigor showed no statistically significant change. After adjustment for multiple comparisons using the Holm procedure, significant reductions remained in Fatigue, Tension–Anxiety, Confusion, and Depression–Dejection. The reduction in Anger–Hostility, although significant in the unadjusted analysis (*p* = 0.030), did not remain statistically significant after adjustment. Vigor was not significantly changed (*p* = 0.354).

**Figure 3:**
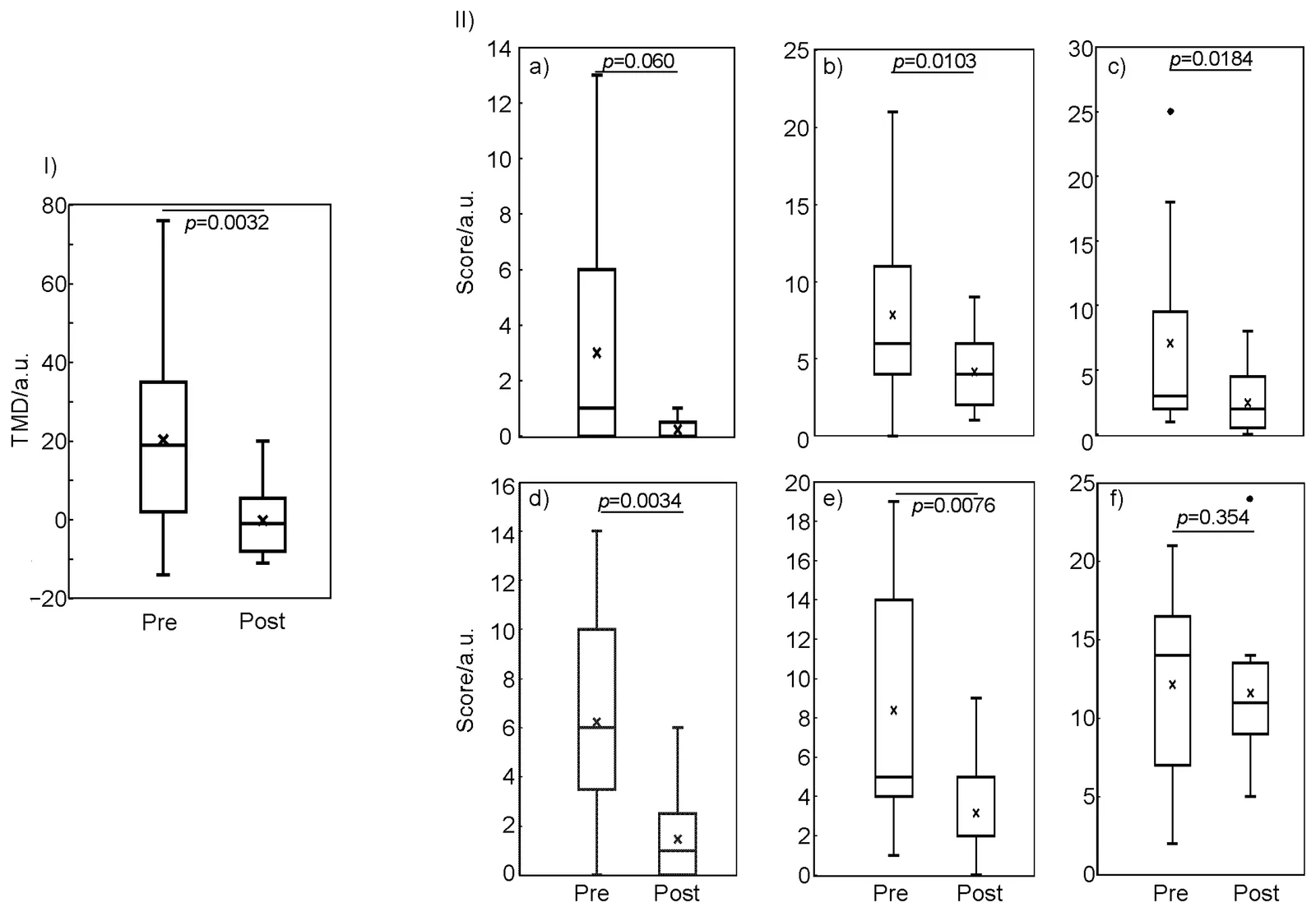
Changes in POMS scores before and after coffee aroma exposure. (**I**) Total Mood Disturbance (TMD); (**II**) six POMS subscale scores before and after exposure to coffee aroma: (**a**) Anger–Hostility; (**b**) Confusion–Bewilderment; (**c**) Depression–Dejection; (**d**) Fatigue–Inertia; (**e**) Tension–Anxiety; (**f**) Vigor–Activity. The *p* value for TMD was obtained using a paired *t*-test. For the six POMS subscales, *p* values shown are Holm-adjusted values for multiple comparisons.

The standardized within-subject effect sizes were 0.58 for Anger–Hostility, 0.98 for Confusion, 0.85 for Depression–Dejection, 1.23 for Fatigue, 1.07 for Tension–Anxiety, and 0.11 for Vigor. Detailed pre- and post-exposure values, mean differences, 95% confidence intervals, unadjusted *p*-values, Holm-adjusted results, and effect sizes are presented in Table 1 and Figure 3.

### 3.3. Change in the EEG-Derived Relaxation Index

**Figure 4:**
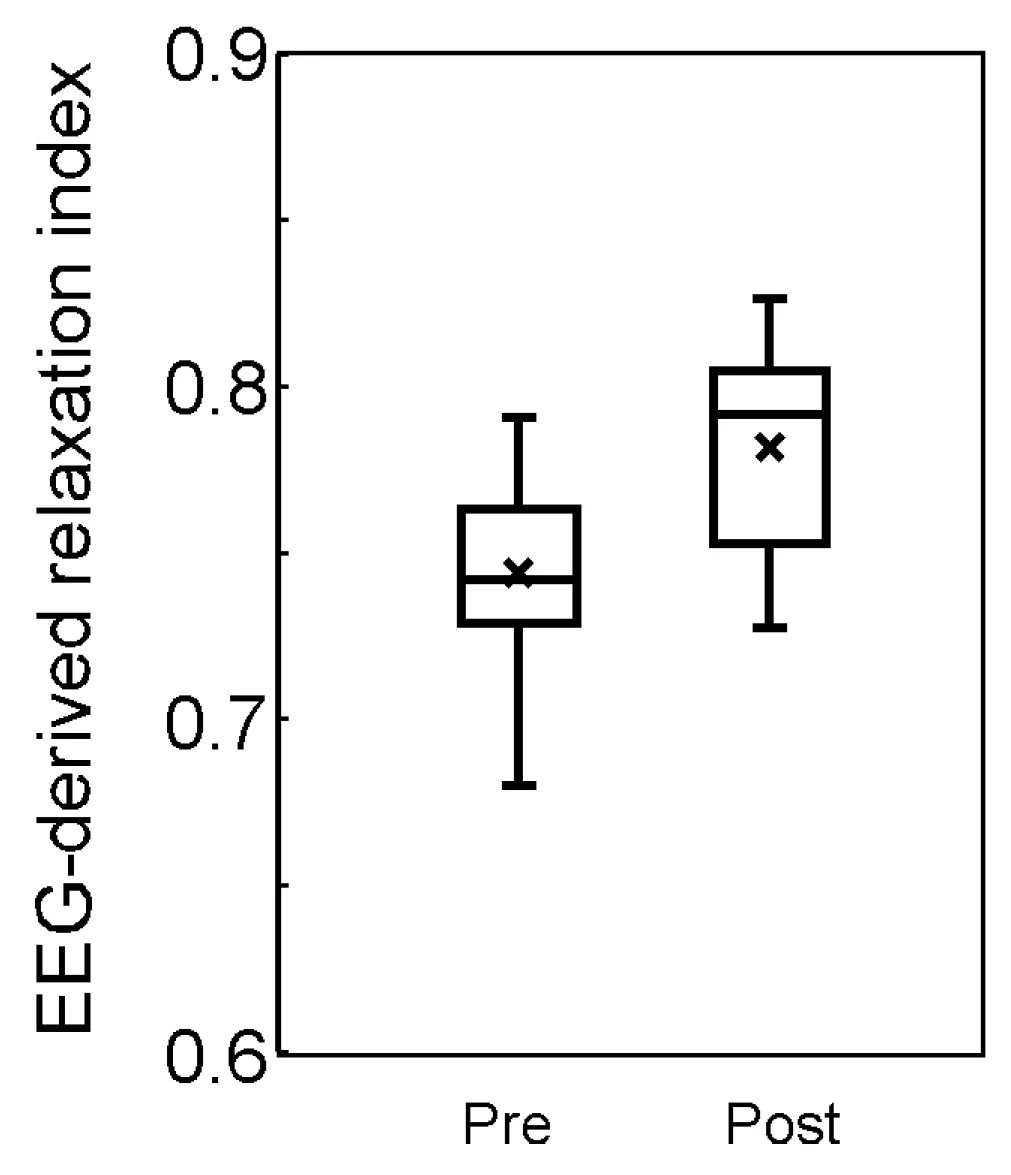
Changes in the EEG-derived relaxation index before and after coffee aroma exposure. The EEG-derived relaxation index was calculated as (α + θ)/(α + β + θ). Boxes represent the interquartile range, horizontal lines within the boxes indicate the median, crosses indicate the mean, and whiskers represent the minimum and maximum values. The index increased significantly after 5 min of coffee aroma exposure (*p* = 0.00025, paired *t*-test).

The EEG-derived relaxation index increased from approximately 0.74 before exposure to 0.78 after exposure. The mean within-subject change was 0.0378 (95% CI: 0.0248–0.0508), with a Cohen’s dz of 1.36 (*p* = 0.00025) (Figure 4).

### 3.4. Changes in Heart Rate and Heart Rate Variability

No statistically significant pre–post change was detected in HR. The mean difference was 1.307 beats/min (95% CI: −1.896 to 4.510), with a Cohen’s dz of 0.19 (*p* = 0.260).

Similarly, no statistically significant change was detected in HRV assessed using the LF/HF ratio. The mean difference was 0.397 (95% CI: −0.990 to 1.784), with a Cohen’s dz of 0.13 (*p* = 0.3255).

**Figure 5:**
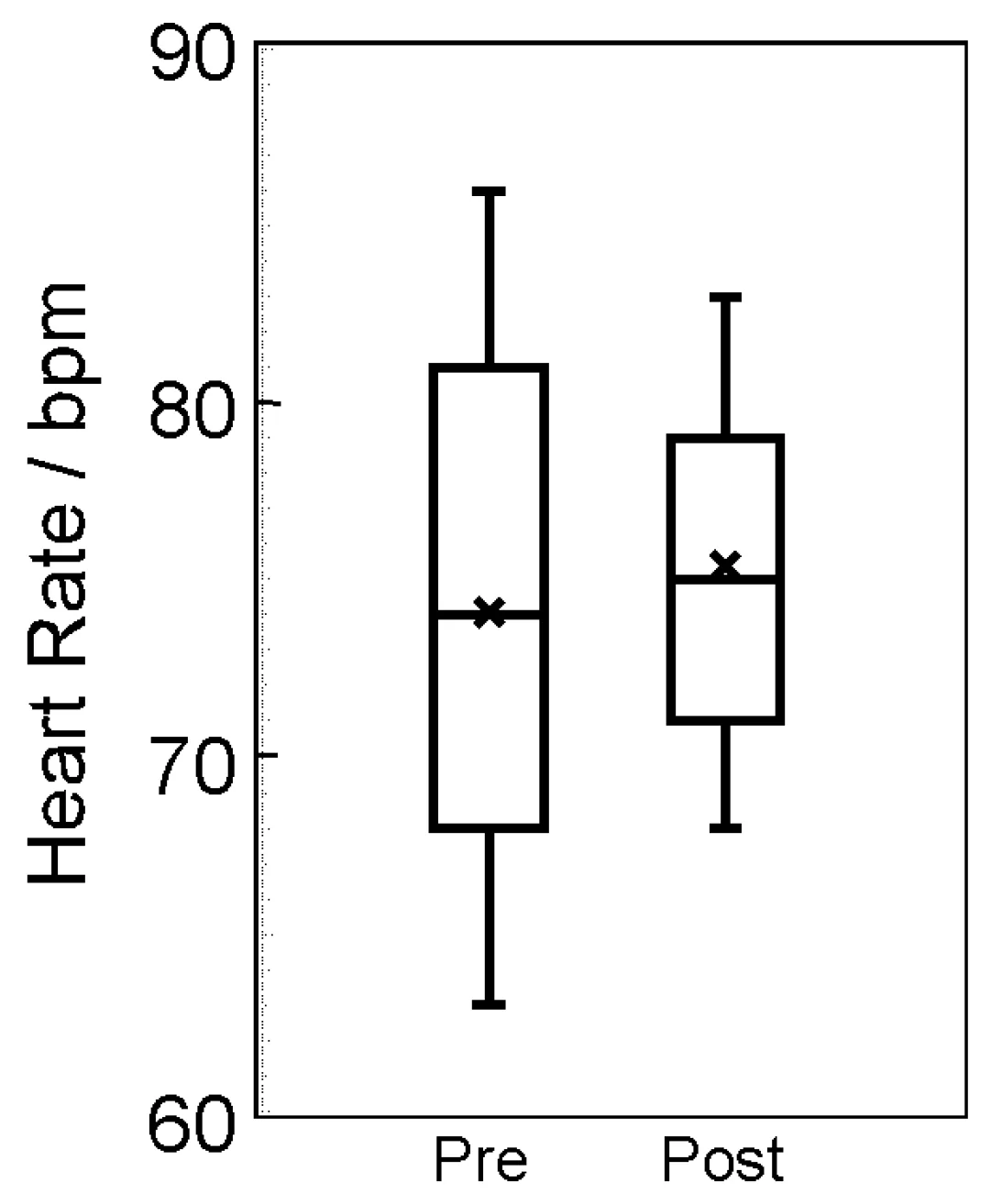
Changes in heart rate before and after coffee aroma exposure. Boxes represent the interquartile range, horizontal lines within the boxes indicate the median, crosses indicate the mean, and whiskers represent the minimum and maximum values. No statistically significant change in heart rate was detected after 5 min of coffee aroma exposure (*p* = 0.260, paired *t*-test).

**Figure 6:**
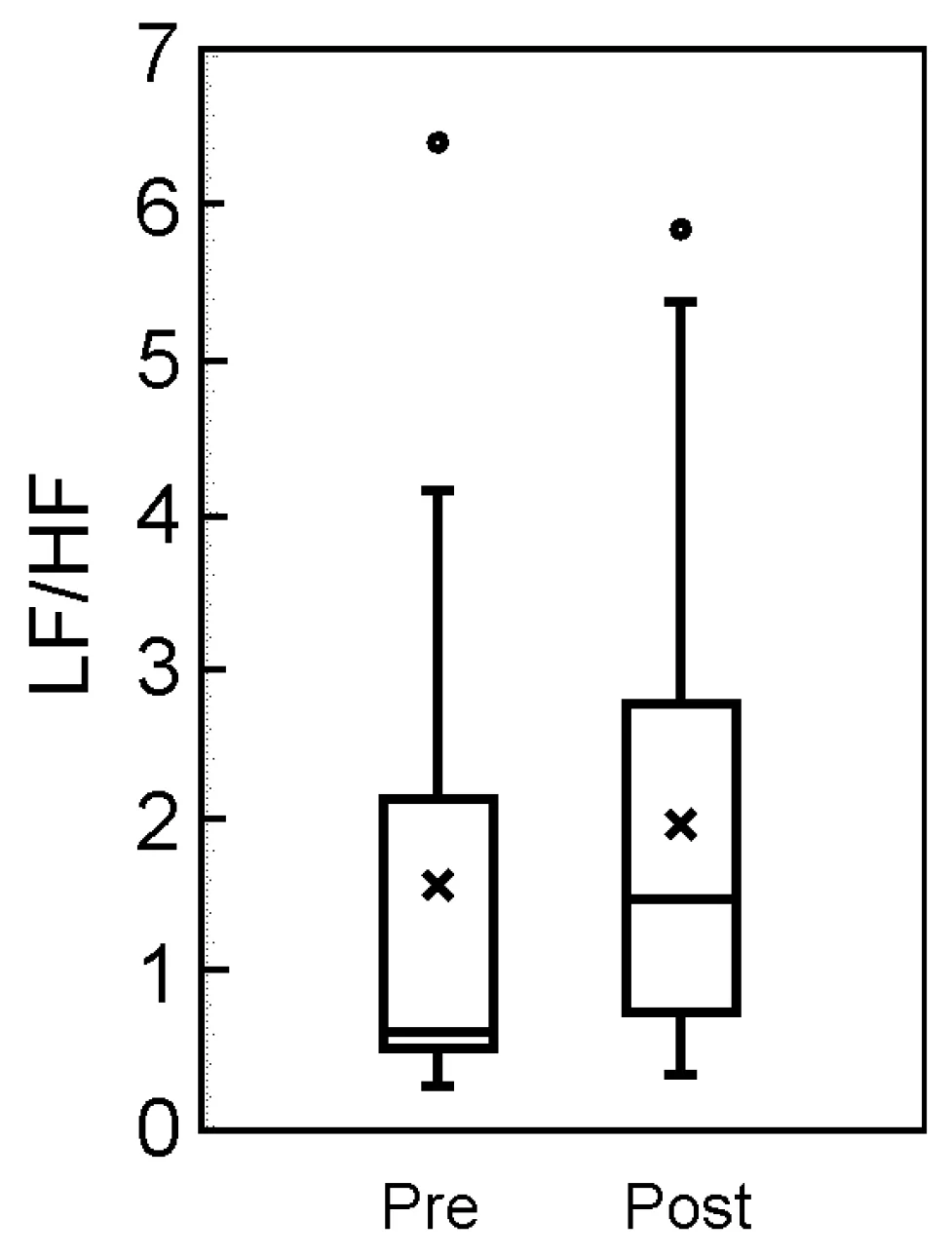
Changes in heart rate variability before and after coffee aroma exposure. Heart rate variability was evaluated using the low-frequency/high-frequency (LF/HF) ratio. Boxes represent the interquartile range, horizontal lines within the boxes indicate the median, crosses indicate the mean, whiskers represent the minimum and maximum values excluding outliers, and circles indicate outliers. No statistically significant change in the LF/HF ratio was detected after 5 min of coffee aroma exposure (*p* = 0.3255, paired *t*-test).

Pre- and post-exposure values for HR and HRV are shown in Figure 5 and Figure 6, respectively.

The pre- and post-exposure values, mean within-subject changes, 95% CIs, *p* values, and effect sizes for the psychological and physiological outcomes are summarized in Table 1.

### 3.5. Exploratory Cross-Domain Associations

Exploratory Pearson correlation analyses were performed to examine individual-level associations among changes in subjective mood, EEG activity, HR, and HRV. No statistically significant correlations were observed between ΔTMD and ΔEEG (r = 0.300, *p* = 0.319), ΔTMD and ΔHR (r = −0.145, *p* = 0.625), ΔTMD and ΔHRV (r = 0.213, *p* = 0.484), ΔEEG and ΔHR (r = 0.466, *p* = 0.108), or ΔEEG and ΔHRV (r = −0.034, *p* = 0.913).

## 4. Discussion

### 4.1. Interpretation of the Findings

The present exploratory within-subject study examined short-term psychological and physiological changes observed following coffee aroma exposure using a multimodal assessment comprising POMS, EEG, HR, and HRV. TMD decreased following the 5 min exposure, and reductions in Fatigue, Tension–Anxiety, Confusion, and Depression–Dejection remained statistically significant after adjustment for multiple comparisons. The EEG-derived relaxation index also increased significantly. In contrast, no statistically significant changes were detected in HR or HRV. Exploratory cross-domain analyses did not identify significant associations among individual changes in subjective mood, EEG activity, HR, and HRV. Taken together, these findings indicate that the different measures provide complementary, rather than necessarily concordant, information regarding the changes observed following coffee aroma exposure.

The observed changes in mood are broadly consistent with previous reports suggesting that coffee aroma can influence subjective psychological states. Hawiset reported that a single exposure to coffee fragrance affected mood and working memory in healthy young adults [4]. The present findings extend this observation by showing reductions in TMD and several negative mood dimensions when mood was evaluated concurrently with cortical and cardiovascular measures. After adjustment for multiple comparisons, reductions remained significant for Fatigue, Tension–Anxiety, Confusion, and Depression–Dejection, whereas the reduction in Anger–Hostility did not remain statistically significant and Vigor showed no significant change. Thus, the observed pattern was characterized primarily by reductions in negative mood dimensions rather than by an increase in positive activation. However, because the present study did not include a control condition, these pre–post changes cannot be attributed specifically to coffee aroma.

The EEG-derived relaxation index increased following coffee aroma exposure. Previous studies have demonstrated that olfactory stimulation can be accompanied by changes in EEG activity, and EEG has been widely used to evaluate psychophysiological responses to fragrances [6]. Park et al. reported changes in quantitative EEG measures during exposure to several fragrances, including coffee aroma [9]. In the present study, the mean increase in the EEG-derived relaxation index was 0.0378 (95% CI: 0.0248–0.0508), with a large standardized within-subject effect size (Cohen’s dz = 1.36). Nevertheless, the absolute magnitude of the change was modest, and the physiological significance of a change in this magnitude in the calculated index has not been established. The EEG finding should therefore be interpreted as a change in the calculated EEG-derived index rather than as definitive evidence that coffee aroma induced a physiologically meaningful state of relaxation.

An important finding of the additional exploratory analyses was that the group-level changes in subjective mood and EEG activity were not accompanied by significant individual-level associations between these measures. Specifically, the change in TMD was not significantly correlated with the change in the EEG-derived relaxation index. Furthermore, neither TMD nor EEG changes were significantly associated with changes in HR or HRV. Thus, the concurrent group-level changes in POMS and EEG should not be interpreted as evidence that the subjective psychological response is directly reflected in the EEG response or that the two share a common underlying mechanism. Rather, these results illustrate the value of assessing multiple domains concurrently: different measures may capture complementary aspects of the response without necessarily changing in parallel within individuals.

No statistically significant changes were detected in HR or HRV following coffee aroma exposure. The standardized within-subject effect sizes were small for both HR (dz = 0.19) and HRV (dz = 0.13), and the 95% confidence intervals for the mean changes included zero. Previous studies of olfactory stimulation have reported variable autonomic responses depending on the odorant and experimental conditions. For example, Sayorwan et al. reported autonomic and EEG changes following lavender inhalation [7], whereas Hawiset found no significant change in heart rate following a single exposure to coffee fragrance [4]. The present HR finding is therefore consistent with the latter observation, although differences in odorants, exposure procedures, study populations, and measurement methods limit direct comparison across studies. Importantly, these non-significant findings should not be interpreted as demonstrating the absence of cardiovascular autonomic responses, particularly given the small sample size and the uncertainty reflected in the confidence intervals.

The differing findings across subjective mood, EEG, HR, and HRV suggest that multimodal assessment may provide complementary information regarding short-term responses following coffee aroma exposure. Differences in measurement characteristics, physiological pathways, or temporal response dynamics could potentially contribute to the lack of correspondence across domains; however, these possibilities were not directly examined and should be regarded as hypotheses for future investigation rather than explanations established by the present data. Importantly, the absence of significant cross-domain correlations indicates that multimodal assessment should not be equated with evidence of an integrated physiological mechanism. Rather, its value in the present study lies in demonstrating that group-level changes across different measures do not necessarily correspond at the individual level.

### 4.2. Strengths and Limitations

The principal strength of the present study is the concurrent assessment of subjective mood, EEG activity, HR, and HRV within the same participants and under the same experimental conditions. This multimodal approach enabled psychological, cortical, and cardiovascular responses to be examined together and, through exploratory cross-domain analyses, allowed their individual-level correspondence to be evaluated.

The present study also has several limitations. The primary limitation is the absence of a control condition. Because no odor-free, placebo, or control-odor condition was included, the observed pre–post changes cannot be attributed specifically to coffee aroma. Time, quiet rest, repeated measurement, expectation, adaptation to the experimental environment, or other nonspecific factors may have contributed to the observed changes. Second, the sample size was small (n = 20), and no formal a priori sample-size calculation was performed. This limits the precision of the effect estimates, particularly for the non-significant HR and HRV findings, and these findings should not be interpreted as evidence of the absence of cardiovascular autonomic responses. Third, the participants were healthy adults aged 20–29 years recruited from university students using convenience sampling, which limits the generalizability of the findings. Fourth, habitual coffee consumption, coffee preference, aroma familiarity, and individual olfactory sensitivity were not systematically assessed. Fifth, although the coffee preparation and exposure procedures were standardized, aroma concentration and airflow were not quantitatively monitored, and room temperature and relative humidity were not systematically recorded; therefore, the intensity of olfactory exposure may have varied among participants. Sixth, only the short-term response to a single 5 min exposure was evaluated. Finally, although the portable EEG device reduced participant burden, consumer-grade wearable EEG systems have technical limitations compared with laboratory-grade EEG equipment [10,11].

### 4.3. Future Perspectives

Future studies should employ randomized controlled designs incorporating an odor-free or appropriate control-odor condition and larger, more diverse samples. Systematic assessment of habitual coffee consumption, coffee preference, aroma familiarity, and olfactory function, together with more precise monitoring of aroma concentration and environmental conditions, would improve the interpretation and reproducibility of the findings. Repeated measurements during and after aroma exposure may also help clarify the temporal relationships among subjective, cortical, and cardiovascular responses.

## 5. Conclusions

In this exploratory within-subject study, short-term coffee aroma exposure was followed by reductions in TMD and several negative mood dimensions and an increase in an EEG-derived relaxation index, whereas no statistically significant changes in HR or HRV were detected. Exploratory cross-domain analyses identified no significant associations among individual changes in subjective mood, EEG activity, and cardiovascular measures, indicating that the concurrent group-level changes should not be interpreted as evidence of a common underlying mechanism. These findings highlight the value of multimodal assessment for characterizing complementary psychological and physiological changes observed following coffee aroma exposure. Controlled studies with larger samples are needed to determine the specificity and physiological significance of these findings.

## Figures and Tables

**Table 1:** Changes in Psychological and Physiological Outcomes Following Coffee Aroma Exposure.

	Pre	Post	Mean			Holm-	Cohen’s
Outcome	Mean ± SD	Mean ± SD	Change	95% CI	Raw *p*	Adjusted *p*	dz
Total Mood	20.40 ± 24.45	−0.20 ±8.97	−20.54	−30.65 to −10.43	0.0032	—	−0.95
Disturbance							
(TMD)							
Anger–	3.00 ± 4.83	0.23 ± 0.44	−2.70	−4.989 to −0.549	0.03	0.06	−0.58
Hostility							
Confusion–	7.85 ± 5.44	4.15 ± 2.34	−3.69	−5.447 to −1.937	0.00258	0.0103	−0.98
Bewilderment							
Depression–	7.08 ± 7.22	2.46 ± 2.60	−4.62	−7.155 to −2.075	0.006125	0.0184	−0.85
Dejection							
Fatigue–	6.23 ± 4.59	1.46 ± 1.76	−4.77	−6.588 to −2.950	0.000562	0.0034	−1.23
Inertia							
Tension–	8.38 ± 5.78	3.15 ± 2.44	−5.23	−7.525 to −2.937	0.00152	0.0076	−1.07
Anxiety							
Vigor–	12.15 ± 5.76	11.62 ± 4.65	−0.54	−2.806 to 1.73	0.354	0.354	−0.11
Activity							
EEG-derived	0.74396 ± 0.029	0.78179 ± 0.029	0.0378	0.0248 to 0.0508	0.00025	—	1.36
relaxation index							
Heart rate (HR),	74.08 ± 6.88	75.38 ± 4.72	1.307	−1.896 to 4.51	0.26	—	0.19
bpm							
HRV (LF/HF)	1.573 ± 1.970	1.970± 1.755	0.397	−0.990 to 1.784	0.326	—	0.13

Note: Data are presented as mean ± SD. Mean change was calculated as post-exposure minus pre-exposure. The 95% confidence intervals (CIs) are for the mean within-subject changes. Cohen’s dz was calculated using the mean and standard deviation of the paired differences. Raw *p* values were obtained using paired *t*-tests. The Holm procedure was applied to the six POMS subscales to account for multiple comparisons; TMD was analyzed separately as an overall mood measure.

## Data Availability

The original contributions presented in the study are included in the article; further inquiries can be directed to the corresponding author.
